# The Metabolic Architecture of Glaucoma: A Unified Framework of Cofactor Failure and Kynurenine Dysregulation

**DOI:** 10.3390/ijms27104311

**Published:** 2026-05-12

**Authors:** Liva Caikovska, Alberts Veitners, Diana Lavrinovica, Juris Vanags, Kristaps Klavins, Guna Laganovska, Arturs Zemitis

**Affiliations:** 1 Department of Ophthalmology, Riga Stradins University, LV-1007 Riga, Latvia; alberts.veitners@gmail.com (A.V.); diaanalavrinovicha@gmail.com (D.L.); jurisvanags@inbox.lv (J.V.); glaganovska@ml.lv (G.L.); arturs.zemitis@gmail.com (A.Z.); 2 Clinic of Ophthalmology, Pauls Stradins Clinical University Hospital, LV-1002 Riga, Latvia; 3 Institute of Biomaterials and Bioengineering, Faculty of Natural Sciences and Technology, Riga Technical University, LV-1048 Riga, Latvia; kristaps.klavins_3@rtu.lv

**Keywords:** glaucoma, metabolomics, aqueous humor, tetrahydrobiopterin, kynurenine pathway, kynurenine monooxygenase, NAD, nicotinamide, one-carbon metabolism, alpha-1 antitrypsin, folic acid, retinal ganglion cells, neuroprotection

## Abstract

Glaucoma remains a primary cause of blindness, yet its pathogenesis often extends beyond intraocular pressure (IOP). This review integrates four converging lines of metabolic evidence—aqueous humor (AH) metabolomics, kynurenine pathway (KP) activity, tetrahydrobiopterin (H4BIP) biology, and NAD/one-carbon dysfunction—into a testable framework for retinal ganglion cell vulnerability. By utilizing a systematic AH metabolomics atlas covering glaucoma, pseudoexfoliation, and diabetes on a standardized HILIC-LC-HRMS platform, we demonstrate that, while aromatic amino acid elevations are non-specific markers, kynurenine monooxygenase (KMO) upregulation is a condition-specific glaucoma signature. These local findings are corroborated by systemic evidence: POAG patients exhibit significant folic acid deficiency (*p* = 0.007) and elevated alpha-1-antitrypsin (AAT). Critically, AAT correlates inversely with both serum folate (r_s_ = −0.485, *p* < 0.001) and retinal nerve fiber layer thickness (r_s_ = −0.386, *p* = 0.017), providing the first in-patient evidence linking systemic inflammation to structural optic nerve damage. We conclude that KMO serves as a critical enzymatic node linking tryptophan metabolism, H4BIP availability, and NAD synthesis. These results characterize glaucoma as a disease of progressive cofactor failure and define a research agenda for multimodal metabolic neuroprotection.

## 1. Introduction

With cases expected to jump by 40% over the next two decades, glaucoma is fast becoming the most significant threat to global vision. It already affects roughly 80 million individuals, a figure projected to reach 111.8 million by 2040 [1]. Managing glaucoma today is a race against optic neuropathy where IOP reduction is our only proven tool [2]. However, the efficacy of this approach is incomplete. Longitudinal data reveal a sobering reality—well-controlled pressure fails to halt progression in nearly half of all patients, highlighting a critical gap in our neuroprotective strategies [3]. Evidence of IOP-independent neurodegeneration necessitates a paradigm shift toward investigating the metabolic “bottlenecks” inherent to RGCs. These neurons represent a unique bioenergetic challenge: their intra-retinal axons lack the energy-saving benefits of myelination, creating a high ATP demand that, when coupled with a lack of regenerative capacity, leaves them acutely vulnerable to homeostatic stress [4,5]. Current IOP-lowering treatments and their principal metabolic and mitochondrial considerations are summarized in Table 1; none directly addresses the metabolic vulnerabilities discussed in this review, motivating investigation of complementary neuroprotective strategies.

Metabolomics—the profiling of small molecules in biological fluids—provides a window into the biochemical state of ocular tissues without direct biopsy [16]. Aqueous humor (AH) is particularly informative, reflecting metabolic activity of the ciliary body, lens epithelium, and trabecular meshwork [17]. A growing body of AH metabolomic studies has identified disruptions in antioxidant defense, amino acid metabolism, neurotransmitter cycling, and energetic substrate utilization across anterior segment diseases, including glaucoma, pseudoexfoliation syndrome (PEXS), and diabetic eye disease [18,19,20]. Other metabolic factors such as glutamate excitotoxicity, sulfur-containing amino acid accumulation, and serine/glycine deficiencies have been identified as key drivers of retinal neurodegeneration and mitochondrial oxidative stress [21]. Systematic reviews confirm that the most consistently replicated AH findings across independent cohorts in glaucoma are arginine/proline pathway enrichment and glutamine/glutamate elevations—implying vascular dysfunction and excitotoxic stress as shared metabolic features [22,23].

Our research group has developed a cross-condition AH metabolomics program at Pauls Stradins Clinical University Hospital, analyzing the same patient population on the same HILIC-LC-HRMS analytical platform across five complementary studies covering glaucoma [24], kynurenine pathway enzyme activity across disease states [25], cataract stage [26], pseudoexfoliation syndrome [27], and diabetes [28]. The HILIC-LC-HRMS platform from Riga Technical University has been applied to peripheral neurodegeneration. Setlere et al. and Klavins used this system to identify metabolite signatures in Charcot–Marie–Tooth disease that correlate with clinical severity. This work validates the platform’s capacity for cross-condition biomarker profiling in Latvian patient populations at Pauls Stradins [29]. Complementing this ocular program, Caikovska et al. [30]—a case-control study of 31 POAG patients versus 26 healthy controls from the same institution—demonstrated significantly lower serum folic acid (*p* = 0.007), higher serum AAT (*p* = 0.028), and critically, a significant inverse correlation between AAT and RNFL thickness (r_s_ = −0.386, *p* = 0.017) and between AAT and folate (r_s_ = −0.485, *p* < 0.001) in the same patients. We propose—as a working hypothesis requiring independent validation—that these converging findings may be connected through H4BIP-dependent enzyme impairment and KMO-mediated kynurenine diversion, with plausible downstream consequences for NAD synthesis and excitotoxic signaling. We emphasize that the AH studies are from a single center and require independent replication.

This framework is further informed by independently replicated lines of evidence: Eichwald et al. [31] established that H4BIP has cytoprotective roles extending beyond classical cofactor function, including direct antioxidant potency, mitochondrial respiratory chain activation, and mitophagy regulation. Williams, Tribble and colleagues demonstrated that age-dependent NAD decline drives RGC bioenergetic vulnerability, confirmed in patient peripheral blood [32,33,34,35,36], and that one-carbon metabolism dysfunction is an early, therapeutically reversible feature of glaucoma [37]. Yuan et al. [38] independently documented elevated plasma AAT in 163 glaucoma patients versus 111 healthy controls, correlating with disease severity. This review synthesizes all frameworks with explicit evidence quality grading, examines alternative explanations, acknowledges limitations, including cohort overlap within our own program, and presents a prioritized research agenda.

## 2. Critical Appraisal of the Primary Metabolomic Evidence

**Targeted AH metabolomics—Zemitis et al. (2026) [24].** Zemitis et al. [24] performed targeted LC-HRMS metabolomic profiling of 53 metabolites in AH from 191 patients: 43 with open-angle glaucoma (16 POAG, 27 PEXG) and 148 cataract-only controls. All metabolites met Level A identification confidence [39]. Tryptophan (FC = 5.89), leucine (FC = 4.60), tyrosine (FC = 5.07), phenylalanine (FC = 3.78), and glutamine (FC = 1.97) were significantly upregulated (all FDR-adjusted *p* = 0.036). The analytical platform—HILIC with seven-point calibration and isotopically labelled internal standards—represents best practice for targeted AH metabolomics [39].

**Kynurenine pathway enzyme activity—Zemitis et al. (2025) [25].** Zemitis et al. [25] measured KP enzyme activity in AH from 191 patients stratified across glaucoma (n = 43), diabetes (n = 55), and PEXS (n = 80). KAT and KMO activities were estimated using metabolite ratios (KYNA/KYN for KAT; 3-HK/KYN for KMO). Glaucoma patients showed significantly elevated KMO activity (*p* = 0.032). KAT activity declined with increasing cataract severity (SPONCS grade, *p* = 0.014). Critically, PEXS showed no significant KP alterations. These findings are from a single center and are best interpreted as hypothesis-generating evidence requiring independent replication.

Central to our research is the Pauls Stradins AH metabolomics atlas, a comprehensive resource derived from a standardized HILIC-LC-HRMS platform. Unlike multi-center meta-analyses, our program utilizes a singular analytical pipeline to facilitate direct comparisons across various anterior segment pathologies. To date, this atlas comprises five investigations into overlapping cohorts, ranging from glaucoma and PEXS to diabetic and cataract-severity groups [24,25,26,27,28]. While the consistency in sample sizes (n ≈ 191) ensures statistical stability across the program, the presence of comorbidities—such as concurrent glaucoma within the diabetes study [28]—introduces a level of “bidirectional contamination.” Acknowledging this overlap is essential when isolating the metabolic signatures unique to a single disease state.

Comparative analysis across these cohorts unveils a metabolic stratification that remains obscured in single-disease studies. While elevations in **phenylalanine, tyrosine, and tryptophan** appear to be conserved, non-specific markers of oxidative stress across glaucoma, PEXS, and diabetes, it is critical to note that these aromatic amino acids and their residues are strong reducers themselves. Consequently, their presence can significantly influence the cellular redox status, potentially acting as a direct modulatory factor in the ocular environment [40,41]. Enzymatic signatures provide a clearer point of divergence. Specifically, **KMO upregulation** serves as a discriminating feature of glaucoma and diabetes. Furthermore, the tryptophan pathway exhibits opposing **metabolic polarities** between the two conditions: glaucoma is characterized by upstream substrate accumulation (tryptophan), whereas diabetes is marked by accelerated downstream consumption toward **3-HK** [24,28]. These localized metabolic shifts are contrasted in Table 2.

Regarding external validation of the cross-condition findings in Table 2: phenylalanine, tyrosine, and glutamine elevations in glaucoma AH are cross-validated across multiple independent platforms and cohorts as confirmed by the Wang et al. [22] and Tang et al. [23] systematic reviews, which identified these metabolites among the 17 most consistently replicated findings across at least three independent studies each. The Sun et al. [21] bidirectional Mendelian randomization study using FinnGen GWAS data (n = 218,792) found no genetically encoded causal signal for aromatic amino acid elevation in glaucoma, further supporting the interpretation that phenylalanine and tyrosine elevations are secondary downstream responses to oxidative stress rather than genetically driven primary metabolic changes—consistent with the non-specific pattern observed across glaucoma, PEXS, and diabetes in Table 2. Tryptophan elevation in glaucoma is not cross-validated externally and is correctly flagged as a single-program finding throughout. KMO enzyme activity elevation in glaucoma has not been replicated on any other platform and should be treated as hypothesis-generating single-center evidence. The PEXS aromatic amino acid findings are consistent with the broader literature on anterior segment oxidative stress in PEXS, including the earlier Laganovska et al. [42] demonstration of elevated serum kynurenine and AH neopterin in PEXS at the same institution. The diabetes 3-HK elevation is consistent with the well-established KP activation in diabetic systemic inflammation [43], though direct independent AH metabolomics replication in diabetic versus non-diabetic cataract patients on other platforms is not yet available.

Methodological limitations applicable across the study program. Several limitations substantially constrain mechanistic inference from these AH datasets. First, all AH study participants underwent cataract surgery, meaning the control groups consisted of cataract patients rather than metabolically healthy individuals. The absence of healthy AH controls is a fundamental limitation of the AH metabolomics field as a whole. Our SPONCS-stratified cataract metabolomics study [26] directly quantified AH metabolite variation as a function of cataract hardness, and the 2025 KP paper [25] demonstrated KAT activity declining significantly with cataract severity (*p* = 0.014)—making this a directly measured internal confound rather than a theoretical concern. Second, concurrent conditions were not uniformly excluded across AH studies, introducing bidirectional contamination between disease groups. Third, all 43 glaucoma patients in the 2026 AH cohort received topical antiglaucoma therapy, while controls received none; beta-blockers reduce AH production by 20–30%, potentially concentrating all amino acids non-specifically, a confound that can only be resolved through treatment-naive patient recruitment. Fourth, H4BIP, H2BIP, neopterin, AH NAD, and quinolinic acid were not measured in any of our AH studies—the H4BIP deficiency hypothesis and the KP-NAD connection, therefore, rest on substrate accumulation patterns and enzyme activity ratios, and these unmeasured variables are the most important gap in the entire evidence base.

Each of the four metabolic frameworks discussed—BH4 deficiency, KP dysregulation, NAD decline, and one-carbon metabolism impairment—converges on retinal ganglion cell bioenergetic and oxidative vulnerability, and each has a corresponding candidate neuroprotective intervention: BH4 restoration via sepiapterin, KMO inhibition to restore kynurenic acid (KYNA) and protect against excitotoxicity, nicotinamide supplementation for NAD repletion and mitochondrial rescue, and B-vitamin cofactor supplementation to support one-carbon metabolism and folate-dependent BH4 recycling.

## 3. The Kynurenine Pathway: A Proposed Mechanistic Bridge

The kynurenine pathway (KP) is the principal route of tryptophan catabolism, accounting for approximately 95% of tryptophan metabolism systemically [44]. The pathway branches at kynurenine: KAT converts kynurenine to KYNA, a broad-spectrum NMDA receptor antagonist with neuroprotective and antioxidant properties; KMO converts kynurenine to 3-hydroxykynurenine (3-HK), a neurotoxic precursor of quinolinic acid and a source of ROS [45]. The KYNA/3-HK balance is, therefore, a critical determinant of whether tryptophan catabolism in the eye is neuroprotective or neurotoxic. Independent preclinical work from other groups shows that optic nerve injury and NMDA-induced RGC death trigger KAT downregulation and transient KMO upregulation in rodent retina, and a murine hereditary glaucoma model shows reduced retinal KP metabolites [46]—findings that provide biological context for our enzymatic observations. Beyond ocular neurodegeneration, systemic metabolic profiling of Latvian cohorts has identified the kynurenine pathway as a key discriminator in peripheral nerve damage [47].

**KMO elevation is condition-specific—the PEXS negative and diabetes contrast.** The single most important argument for the specificity of the glaucoma KMO finding emerges from the cross-condition atlas. PEXS patients show elevated phenylalanine, tyrosine, and tryptophan in AH [24]—consistent with oxidative stress and inflammatory activation—yet show no significant KP enzyme changes in the same cohort [25]. This means aromatic amino acid accumulation alone is not sufficient to produce KMO upregulation in AH. KMO elevation in glaucoma is not simply a passive consequence of elevated tryptophan substrate; it is a distinct, condition-specific enzymatic response that may reflect neurodegeneration-specific inflammatory signaling absent in PEXS alone. The contrast with diabetes further strengthens this argument: the diabetes AH metabolomics study [28] found elevated 3-HK and activated tryptophan catabolism pathways in diabetic AH—consistent with KMO upregulation driven by diabetic inflammatory activation—but the direction of tryptophan change is opposite. Diabetes shows elevated 3-HK (substrate consumed, product elevated—KMO saturation), while glaucoma shows elevated tryptophan (substrate accumulated upstream—consistent with H4BIP-dependent TPH impairment limiting tryptophan entry into the KP). This directional divergence in the KP metabolite fingerprint, measured on the same platform in overlapping patient populations, is among the most compelling within-platform evidence supporting the mechanistic specificity of the glaucoma H4BIP-TPH hypothesis. It is, however, a hypothesis generated by cross-condition comparison and not yet tested directly.

**Observations and limitations of the KMO elevation finding.** Our 2025 KP study [25] found significantly elevated KMO activity in glaucoma AH (*p* = 0.032, n = 43 glaucoma patients). This finding is from a single center, enzyme activities were estimated from metabolite ratios rather than measured directly, the study was not powered for POAG versus PEXG subgroup analysis, and the cataract confound described in Section 2 applies here. The absence of KP changes in PEXS [25] suggests the KMO elevation is associated with the glaucomatous process rather than purely PEXS-specific pathology, but this interpretation should be treated as hypothesis-generating pending replication in independent POAG-only cohorts with SPONCS-matched controls.

**A proposed model for cross-study tryptophan discordances.** A plausible mechanistic model can reconcile the apparent contradiction between elevated tryptophan in our 2026 glaucoma cohort [24] and elevated downstream kynurenine in a separate multi-omics study [48]. If H4BIP-dependent TPH activity is impaired in glaucoma, tryptophan accumulates upstream regardless of KMO activity, because less tryptophan enters the serotonin route [49]. Simultaneously, KMO upregulation driven by neurodegeneration-associated inflammation would consume available kynurenine toward 3-HK, preventing kynurenine accumulation despite elevated upstream tryptophan. In diabetes, where TPH impairment is less likely the primary driver, tryptophan enters the kynurenine route normally, and elevated KMO fully converts it to 3-HK. We acknowledge this model is proposed rather than demonstrated, and that cohort heterogeneity, medication effects, and cataract severity variation remain equally plausible alternative explanations.

**The proposed KP-NAD connection.** The kynurenine pathway is the primary endogenous route for de novo NAD synthesis from tryptophan via quinolinic acid. Elevated KMO activity would, if confirmed, divert kynurenine flux toward 3-HK and ROS rather than toward quinolinic acid and NAD—a plausible locally ocular mechanism for NAD precursor supply impairment, complementing the well-established systemic NAD precursor deficiency documented by Kouassi Nzoughet et al. [50] and the retinal NAD decline demonstrated by Williams et al. [33]. In diabetes, elevated 3-HK in AH [28] is consistent with this diversion already occurring in the diabetic ocular compartment. We have not directly measured AH NAD concentrations or quinolinic acid in any of our studies; this connection is inferential and is presented as a research priority motivating future direct measurement.

**Reduced KYNA and the proposed amplification of excitotoxicity.** KMO upregulation, reducing available kynurenine for KAT-mediated KYNA synthesis, would diminish endogenous NMDA receptor antagonism. This compounds the documented AH and vitreous glutamate/glutamine elevation [51,52] to create a compounding excitotoxic burden on RGCs. This mechanism is plausible and consistent with independent data but requires direct KYNA quantification in treatment-naive glaucoma AH to be considered established.

## 4. Alternative Explanations for AH Amino Acid Elevations

Three alternative explanations for the aromatic amino acid elevations observed in our 2026 glaucoma cohort must be considered alongside the H4BIP hypothesis, and the cross-condition atlas data help calibrate their relative plausibility.

**Pharmacokinetic concentration effects.** Beta-adrenergic antagonists reduce AH production by 20–30%, potentially concentrating all amino acids through reduced dilution—a non-specific mechanism that could account for some or all amino acid elevations without invoking any change in metabolism. This is the most parsimonious confound and the most important to address through treatment-naive cohort designs.

**Non-specific anterior segment oxidative stress.** The cross-condition atlas shows phenylalanine, tyrosine, and tryptophan elevations shared across glaucoma and PEXS [24,28], both characterized by anterior segment oxidative stress and elevated TGF-β [53]. ROS directly inhibit H4BIP-dependent hydroxylase activity and B6-dependent aminotransferases [37], concentrating their substrates regardless of disease-specific mechanisms. The cross-condition analysis reveals which elevations are non-specific (aromatic amino acids) versus potentially condition-specific (KMO ratio in glaucoma but not PEXS).

**Vitamin B6-dependent pathway dysfunction.** Tribble and Williams et al. [37] directly demonstrated transcriptomic dysregulation of B6-utilizing enzymes in glaucoma retina and optic nerve head across multiple cell types, including human iPSC-derived RGCs—providing an independent alternative explanation for aromatic amino acid accumulation, unrelated to H4BIP.

Assessing the relative plausibility of these alternatives: the H4BIP hypothesis offers the most parsimonious single explanation for the simultaneous elevation of three substrates processed by entirely different H4BIP-dependent enzymes (PAH, TH, and TPH), and gains additional support from the tryptophan-3-HK directional contrast between glaucoma and diabetes. The finding of elevated tyrosine alongside elevated phenylalanine is inconsistent with simple PAH deficiency—which depletes tyrosine in phenylketonuria—and instead requires simultaneous TH impairment, consistent with global H4BIP insufficiency rather than isolated single-enzyme dysfunction. Nevertheless, the medication confound, the non-specific nature of aromatic amino acid elevation across conditions, and the absence of direct H4BIP measurement mean no mechanistic claim is definitive. The most important next step remains direct H4BIP/H2BIP ratio measurement in treatment-naive POAG patients.

## 5. Tetrahydrobiopterin Deficiency: A Working Hypothesis in Glaucoma

We present H4BIP deficiency as a working hypothesis—internally consistent, mechanistically well grounded, and supported by indirect cross-condition AH evidence—but explicitly not demonstrated in human glaucoma tissue owing to the absence of direct H4BIP or H2BIP measurement in any published AH study, including our own.

**Classical enzymatic roles and NOS uncoupling.** H4BIP (6R-L-erythro-5,6,7,8-tetrahydrobiopterin) is an obligatory cofactor for PAH, TH, TPH, all three NOS isoforms, and alkylglycerol monooxygenase [31,54]. For NOS, H4BIP stabilizes the enzyme dimer and maintains coupled NO synthesis; when H4BIP:H2BIP ratios fall—due to oxidative consumption or impaired synthesis/salvage—NOS uncouples, generating superoxide (O_2_•^−^) and peroxynitrite (ONOO^−^), which further oxidize H4BIP in a self-amplifying cycle [54,55]. H4BIP homeostasis depends on de novo synthesis via GTPCH I (encoded by GCH1, the rate-limiting enzyme), recycling via PCD and DHPR, and salvage via DHFR [56]. The arginine/proline pathway—the most consistently replicated metabolomic signal across independent glaucoma cohorts—provides cross-study metabolomic support for NOS/NO pathway dysregulation consistent with, though not specific to, the H4BIP-NOS uncoupling mechanism. A further line of support comes from Baumane et al. [57] at Pauls Stradins, who measured NT-proANP—the stable circulating surrogate for atrial natriuretic peptide (ANP) production—in both plasma and AH of 58 POAG patients undergoing trabeculectomy versus 32 cataract controls, finding significantly elevated NT-proANP in both compartments (plasma: 7.00 vs. 4.65 nmol/L, *p* = 0.005; AH: 0.47 vs. 0.09 nmol/L, *p* = 0.011). ANP activates eNOS through a cGMP-mediated pathway to produce NO—a process requiring H4BIP as cofactor for coupled eNOS function. In H4BIP-deficient conditions, ANP-stimulated eNOS would produce superoxide rather than NO, converting a compensatory vasodilatory signal into an additional oxidative stress. The absence of correlation between AH and plasma NT-proANP in POAG suggests local ocular ANP production or trapping rather than passive plasma equilibration, indicating active anterior segment vascular stress signaling independent of systemic cardiovascular status. Trabeculectomy patients represent more advanced disease than the cataract-surgery cohorts in our metabolomics program, limiting direct magnitude comparison.

**Non-canonical H4BIP roles: direct antioxidant, mitochondrial activator, and mitophagy regulator.** Eichwald et al. [31] established three non-canonical H4BIP roles of direct relevance to RGC biology. First, H4BIP scavenges superoxide, hydroxyl radicals, thiyl radicals, and peroxynitrite more effectively than ascorbate at physiological blood pH—making it an active frontline antioxidant consumed in radical neutralization rather than merely a passive cofactor. In the oxidatively stressed environment of glaucoma, where AH glutathione is consistently reduced [58], simultaneous direct radical consumption and impaired H2BIP-to-H4BIP recycling via GSH-dependent DHFR [55] creates compounding antioxidant vulnerability. Second, H4BIP reduces ferricytochrome c to ferrocytochrome c at the Complex III/IV interface of the respiratory chain; in cells lacking GCH1, mitochondrial respiration was impaired and rescued by sepiapterin supplementation, demonstrating a direct H4BIP-mitochondria connection independent of NOS [31]. Third, H4BIP deficiency impaired mitophagy across multiple tissues in SPR-null mice, causing accumulation of damaged mitochondria [31]. These roles are established in non-ocular models and await direct testing in retinal or AH tissue; their relevance to RGCs is plausible given that mitochondrial dysfunction precedes neurodegeneration in glaucoma [33], but extrapolation should be made cautiously.

A mechanistic feature of H4BIP biology, particularly relevant to the ocular compartment—and that has received insufficient attention in the glaucoma literature—is the rapid autoxidation of H4BIP in the presence of molecular oxygen. H4BIP undergoes spontaneous oxidation to H2Bip and further degradation products under aerobic conditions, with reaction rates accelerated by elevated H_2_O_2_, superoxide, and peroxynitrite [41,59,60]—all present in the oxidatively stressed glaucomatous AH. This creates a self-amplifying oxidative cycle that extends beyond NOS uncoupling: oxidative stress accelerates H4BIP autoxidation to H2Bip, which impairs NOS coupling, which generates further superoxide and peroxynitrite, which further accelerates H4BIP autoxidation. Importantly, pterin products of H4Bip autoxidation—including dihydropterin, dihydroxanthopterin, and pterin—predominate over biopterin products [41], and these oxidized pterins can photosensitize the production of singlet molecular oxygen and thus amplify oxidative stress further [60,61]. The photochemical dimension is specifically relevant to the anterior segment: unlike most body compartments, AH is directly exposed to incident light [61,62], meaning that H2Bip and pterin photooxidation products accumulate in the glaucomatous AH in ways that do not occur systemically. This implies that the H4BIP:H2Bip ratio in AH may be substantially more depleted than systemic plasma measurements would predict, and that any estimate of H4BIP status derived from plasma underestimates the degree of ocular H4BIP insufficiency. For therapeutic purposes, H4BIP generated from sepiapterin via the salvage route would be subject to the same autoxidation as endogenous H4BIP unless the broader oxidative AH environment is simultaneously addressed.

**Neopterin as a candidate biomarker and cytoprotective molecule.** Neopterin—the GTP metabolite upstream of H4BIP in the de novo synthesis pathway—activates Nrf2/ARE, increases mitochondrial number in sensory neurons, and facilitates long-term potentiation in rodent and human neural cells at physiological concentrations [31,63]. In glaucoma, neopterin measurement in AH would provide both a marker of compensatory GTPCH I upregulation and potentially a measure of endogenous neuroprotective activity. Neopterin has not been measured in any published glaucoma AH study; its measurement is a priority for future integrated profiling.

Excessively elevated H4BIP causes mitochondrial dysfunction, immune hyperactivation, and worsening of chronic inflammatory conditions [64]. More specifically, Schallreuter et al. [62,65] demonstrated that H_2_O_2_ accumulation impairs 4a-carbinolamine dehydratase (PCD) recycling of H4BIP, leading to the accumulation of the non-enzymatic 7-isomer of H4BIP (7H4BIP), which acts as a potent competitive inhibitor of PAH with an apparent Ki = 10^−6^ M—leading directly to phenylalanine accumulation [62,65]. Further work confirmed that the 7(S)-H4BIP diastereomer specifically is the competitive inhibitor of PAH [66]. This mechanism is directly relevant to the oxidatively stressed glaucomatous AH: H_2_O_2_ accumulation in the anterior segment could impair PCD recycling, generating 7H4BIP that competitively inhibits PAH and worsens phenylalanine accumulation independently of absolute H4BIP deficiency. This means the relationship between H4BIP metabolism and aromatic amino acid accumulation in glaucoma AH is non-linear and bidirectional, not simply “H4BIP deficiency → substrate accumulation”, and that excessive H4BIP supplementation could paradoxically worsen phenylalanine accumulation through this route. For therapeutic purposes, H4BIP:H2Bip ratio monitoring is, therefore, an essential pharmacodynamic endpoint in any future supplementation study rather than simply measuring total pteridine content [67].

## 6. NAD Decline and Nicotinamide Neuroprotection: The Best-Evidenced Metabolic Framework

In contrast to the H4BIP deficiency and KP bridge hypotheses—which are well grounded but unproven working hypotheses—the NAD/nicotinamide framework has the strongest and most independently replicated evidence base for metabolic neuroprotection in glaucoma. We present this framework first among the therapeutic evidence precisely because of its superior evidentiary standing.

Williams et al. [33] demonstrated that mitochondrial dysfunction and metabolic abnormalities in DBA/2J mouse RGCs precede detectable neurodegeneration—establishing metabolic insufficiency as a predisposing condition rather than a downstream consequence. Retinal NAD levels decline age-dependently [68]. Nicotinamide was neuroprotective at doses producing no IOP change, with approximately 93% of eyes protected at the highest dose—a roughly 10-fold reduction in glaucoma risk replicated in independent rodent models [33]. Gustavsson et al. [69] demonstrated that nicotinamide prevents retinal capillary dropout in rat OHT and supports ocular blood supply in a prospective human clinical cohort, extending protection to the retinal vasculature.

Kouassi Nzoughet et al. [50] demonstrated that plasma nicotinamide was significantly lower in POAG versus age-matched controls (−30%, *p* = 0.022; independently replicated, −33%, *p* = 0.011)—the first direct measurement of NAD precursor deficiency in human POAG. Petriti et al. [32] in Nature Medicine found a lower PBMC mitochondrial oxygen consumption rate (OCR) and cellular NAD in 168 POAG/NTG patients versus 48 controls, with lower OCR independently predicting faster visual field progression (13% of variance, comparable to IOP’s contribution in an untreated reference cohort). Phase II clinical trials demonstrate improvements in inner retinal function [70] and visual field sensitivity [71]. The NAMinG Phase III trial (NCT05405868) is ongoing.

The Petriti/Williams group’s work establishes NAD decline through retinal depletion and systemic NAD precursor deficiency. Our AH KP enzyme data [25] raise the additional, currently inferential hypothesis that elevated KMO activity may locally divert the kynurenine-to-NAD synthesis route in the eye itself, creating a second level of NAD precursor supply impairment that is disease-active and ocular-specific rather than age-related and systemic. In diabetes, elevated 3-HK in AH [28] is consistent with this diversion already occurring in the diabetic ocular compartment. Whether glaucoma-specific KMO upregulation produces a measurable local NAD synthesis deficit requires direct AH quinolinic acid and NAD measurement—these are presented as the highest-priority unmeasured variables in our research program.

## 7. One-Carbon Metabolism Dysfunction in Glaucoma

Tribble, Wong, Stuart et al. [37] provided direct experimental evidence that one-carbon metabolism is disrupted early and sustainedly in glaucoma: transcriptomic dysregulation of B6, B9, B12, and choline-utilizing enzymes across the whole retina, optic nerve head, RGCs, microglia, and infiltrating monocytes—present before neurodegeneration and confirmed in iPSC-derived RGCs from POAG patients. B6/B9/B12/choline cocktail supplementation provided complete RGC neuroprotection in a chronic mouse model independently of IOP. Mendelian randomization in up to 216,257 UK Biobank participants found no significant effect of genetically elevated homocysteine on POAG outcomes [37], redirecting the therapeutic target from homocysteine reduction to broad one-carbon cofactor utilization support.

The epidemiological support is substantial: higher blood folate associates with reduced glaucoma risk [72]; MTHFR A1298C polymorphism associates with glaucoma risk across 42 studies [73]; and alcohol, which impairs folate metabolism [74] and associates with higher PEXG risk [75]. One-carbon metabolism and H4BIP salvage share DHFR as a common enzymatic node—dysregulated B9 utilization would plausibly impair H4BIP regeneration from H2BIP, creating a biochemical co-dependency between the one-carbon and H4BIP frameworks that is mechanistically coherent but unmeasured in glaucoma ocular tissue [76].

The most important new development since prior versions of this review is the direct biochemical serum confirmation from our group. Caikovska et al. [30] measured serum folic acid in 31 POAG patients versus 26 healthy controls and found significantly lower folate in glaucoma patients (median 6.8 vs. 8.96 ng/mL, *p* = 0.007). This is the methodologically cleanest folate measurement available from our program, and the finding converges with the Tribble/Williams transcriptomic data [37] and the epidemiological associations [72,73] to produce three independent lines of evidence for folate deficiency in glaucoma from three different methodologies. Critically, the significant inverse correlation between serum AAT and serum folate in the same patients (r_s_ = −0.485, *p* < 0.001) [30] is consistent with the proposed shared inflammatory upstream driver simultaneously consuming folate while driving AAT acute-phase production—an in-patient observation that cannot be derived from either biomarker measured alone.

## 8. Cross-Study Metabolomic Evidence: Consensus, Discordances, and Emerging Pathways

Tang et al. [23] identified aminoacyl-tRNA biosynthesis and arginine/proline metabolism as the most consistently enriched pathways across AH and plasma in POAG, with D-glutamine/D-glutamate metabolism as the second most enriched AH pathway. Wang et al. [22] identified 17 cross-validated metabolites across at least three independent studies, including phenylalanine, tyrosine, arginine, glutamine, and methionine. Phenylalanine and tyrosine appear in this cross-validated list and in our glaucoma, PEXS, and diabetes datasets—though the cross-condition atlas now contextualizes them as non-specific oxidative stress markers rather than glaucoma-specific signals. Methionine—the downstream product of folate-dependent homocysteine remethylation—provides metabolomic corroboration of the one-carbon framework [37] from independent datasets. The glutamine/glutamate enrichment is the most robust cross-study finding, further supported by the MISO II study [51] showing glutamine and alpha-ketoglutarate predicting glaucoma progression longitudinally.

The apparent contradiction between elevated tryptophan in our glaucoma cohort [24] and elevated kynurenine in a multi-omics study [48] reflects genuine biological heterogeneity between cohorts, disease subtypes, and inflammatory states. The cross-condition data provide a plausible model, but alternative explanations, including subtype differences, medication effects, and cataract severity variation, remain equally plausible. Golpour et al. [20] confirmed arginine, proline, and glutamate as the most consistent plasma findings across studies, further underscoring that aromatic amino acid changes have weaker cross-study support than the NO/arginine and glutamate pathways.

Among emerging systemic biomarkers, AAT has the strongest evidence base. Yuan et al. [38] demonstrated significantly elevated plasma AAT in 163 glaucoma patients versus 111 healthy controls (*p* < 0.001), correlating with disease severity across two grading systems (early versus severe: AUC = 0.763). Caikovska et al. [30] independently confirmed elevated serum AAT in 31 POAG versus 26 healthy controls (mean 1.55 vs. 1.38 g/L, *p* = 0.028, d = 0.617), alongside lower folate, with AAT correlating inversely with both RNFL thickness (r_s_ = −0.386, *p* = 0.017) and serum folate (r_s_ = −0.485, *p* < 0.001). The direction of systemic AAT change—elevated rather than depleted—is consistent with hepatic compensatory upregulation as an acute-phase response to peripheral AAT oxidative inactivation by peroxynitrite generated under H4BIP-deficient NOS uncoupling [53,70]. The co-occurrence with folate deficiency is mechanistically coherent: the same pro-inflammatory cytokines (IL-6, IL-1β, TNF-α) that drive AAT acute-phase production also induce KMO [44] and increase folate consumption [37]. Future serum studies measuring AAT should include SERPINA1 genotyping to exclude carriers of deficiency alleles who may show paradoxically elevated AAT due to inflammatory upregulation masking a genetically low baseline [77]. AAT measurement in AH has not yet been performed in any published study; this represents an important future research priority.

Other emerging findings include the following: NT-proANP (N-terminal pro-atrial natriuretic peptide) significantly elevated in both plasma and AH of POAG patients versus cataract controls from Pauls Stradins—with AH levels showing no correlation with plasma concentrations, consistent with local ocular ANP production or trapping rather than passive diffusion [57]; agmatine and thiamine significantly reduced in POAG AH with protective effects in neuroinflammation models [19]; serum androstenedione elevated in primary angle-closure glaucoma (PACG) correlating with visual field severity and predicting progression [78]—a finding from PACG not yet tested in open-angle glaucoma, but mechanistically coherent through androgen-mediated eNOS suppression; elevated histamine and octanoylcarnitine alongside 3-HK in diabetic AH [28], with putrescine—a neuroprotective polyamine—significantly reduced, consistent with the broader polyamine deficiency pattern identified in glaucoma plasma by Leruez et al. [79] and suggesting shared polyamine vulnerability across ocular neurodegeneration and metabolic eye disease.

## 9. Discussion

This review has developed a testable conceptual framework proposing that the kynurenine pathway may serve as a plausible mechanistic connection linking tryptophan metabolism, H4BIP deficiency, NAD decline, and excitotoxic neurodegeneration in glaucoma. The central strength of this synthesis is its internal coherence: the AH metabolomic findings, the direct KP enzyme observations, the H4BIP cytoprotective biology, and the independently replicated NAD/one-carbon frameworks are mutually consistent and biochemically connected. The central weakness is that the most novel elements derive from a single program at a single center, and the key intervening variables—H4BIP, AH NAD, quinolinic acid, neopterin—have not been directly measured in human glaucoma AH by any group.

The cross-condition AH metabolomics atlas is one of the most important methodological contributions of this synthesis. By comparing glaucoma, PEXS, diabetes, and cataract-stage variation on the same platform, we can now make a distinction that was not possible from any single study: aromatic amino acid elevations are non-specific anterior segment responses to oxidative stress, present across conditions; KMO upregulation is present in glaucoma and diabetes but absent in PEXS; and the direction of tryptophan pathway change is opposite between glaucoma (substrate accumulation) and diabetes (3-HK product elevation). This cross-condition fingerprinting elevates the specificity of the KMO finding and provides the first within-platform evidence distinguishing glaucomatous from diabetic KP dysregulation. The integrated model generates specific falsifiable predictions: H4BIP and H2BIP should show an altered ratio in glaucoma AH; KMO elevation should correlate with reduced quinolinic acid and AH NAD-pathway intermediates; serum AAT and B9 should correlate with AH KP enzyme ratios within the same patient; and combination supplementation of nicotinamide with B-vitamins should be more neuroprotective than either alone.

The published serum study makes a contribution that the AH program alone cannot: using genuinely healthy controls, it provides the cleanest glaucoma versus non-glaucoma comparison for systemic biomarkers. The inverse correlation between circulating AAT and RNFL thickness (r_s_ = −0.386, *p* = 0.017) is the first direct link in a human cohort between this systemic inflammatory biomarker and a validated structural measure of RGC loss. More mechanistically informative still is the inverse correlation between serum AAT and serum folate in the same patients (r_s_ = −0.485, *p* < 0.001): these two biomarkers are anti-correlated within individual patients, which is exactly what the inflammatory unification model predicts—the same cytokine environment driving AAT upregulation simultaneously consuming folate. This in-patient anti-correlation provides the strongest available evidence for a shared upstream inflammatory driver and motivates the prospective integrated profiling study our group is currently preparing. The inflammatory unification model proposes that a low-grade but sustained systemic inflammatory state in glaucoma simultaneously drives KMO upregulation in AH (via IL-6/IL-1β/TNF-α induction of KMO), compensatory H4BIP synthesis followed by oxidative consumption, folate depletion from increased one-carbon utilization by activated immune cells, and peripheral AAT oxidative inactivation with compensatory hepatic upregulation. Its direct test requires simultaneous measurement of cytokines, AAT, folate, H4BIP, and KP metabolites in the same patients.

All therapeutic strategies discussed in this review—nicotinamide, B-vitamin supplementation, sepiapterin, and KMO inhibition—should be understood as experimental strategies requiring controlled clinical trials before they can be recommended in clinical practice. Nicotinamide has Phase II human data and an ongoing Phase III trial and represents the most evidence-supported strategy; B-vitamin supplementation has strong preclinical evidence, direct B9 deficiency serum confirmation, and an established safety profile but awaits clinical trial validation; sepiapterin and KMO inhibition remain early-stage research hypotheses with no published ocular in vivo data.

The most urgent unanswered questions that arise from this synthesis can be grouped into three tiers by immediacy and feasibility. The highest priority is integrated AH and serum profiling combining BH4, H2Bip, neopterin, KYNA, 3-HK, kynurenine, quinolinic acid, NAD-pathway intermediates, B-vitamin cofactors, AAT and its oxidized forms, selenium, and pro-inflammatory cytokines (IL-6, IL-1β, and TNF-α) in treatment-naive, condition-stratified patients—using SPONCS-matched cataract controls and the validated HPLC electrochemical methods now available for small-volume pteridine quantification [80]. This single study design would simultaneously test the BH4, KP, NAD, AAT, and inflammatory unification hypotheses and should be prioritized above any individual measurement in isolation. The second tier comprises independent external replication of the KMO elevation finding in POAG-only cohorts at other centers, and medication-stratified prospective AH collection from treatment-naive patients to resolve the pharmacokinetic concentration confound—both prerequisites before any single-center AH metabolomics finding can be treated as established rather than hypothesis-generating. The third tier—KMO inhibitor animal studies, subtype-stratified metabolomics, AAT measurement in AH, SERPINA1 genotyping in serum studies, longitudinal biomarker-progression correlations, including whether baseline serum AAT and folate predict rates of structural progression, combination supplementation preclinical trials, extension of the cross-condition atlas to NTG and PACG, and serum calcium and bilirubin as prognostic biomarkers from the Sun et al. MR framework [21]—represents the broader research program following confirmation of the core hypotheses. The phase III NAMinG trial and the four other concurrent multi-center nicotinamide trials represent the most immediately actionable clinical research already underway; the metabolic framework presented here predicts that patients with low baseline NAD precursors, low folate, and elevated AAT will be the subgroup with the greatest response to nicotinamide supplementation, and pre-specified subgroup analyses along these biomarker lines would substantially increase the translational value of these trials without requiring additional participants.

## 10. Conclusions

This review has proposed a testable conceptual framework in which the kynurenine pathway may serve as a mechanistic bridge linking four converging metabolic vulnerabilities in glaucoma: aromatic amino acid accumulation consistent with H4BIP-dependent hydroxylase impairment, KMO-mediated diversion of tryptophan catabolism toward neurotoxic 3-HK and away from neuroprotective KYNA and NAD synthesis, H4BIP deficiency driving NOS uncoupling and mitochondrial dysfunction, and systemic NAD decline and one-carbon metabolism impairment. A cross-condition AH metabolomics atlas, covering glaucoma, PEXS, diabetes, and cataract-stage variation on the same analytical platform, reveals that aromatic amino acid elevations are non-specific, while KMO upregulation—absent in PEXS and opposite in direction to the tryptophan pathway change observed in diabetes—is condition-specific. This cross-condition specificity evidence is the most novel methodological contribution of the current synthesis.

The evidence quality hierarchy has strengthened materially across versions of this review. The NAD/nicotinamide and one-carbon frameworks have the strongest, most independently replicated evidence, including direct human biomarker data from multiple groups. Systemic AAT elevation has been independently replicated across two published cohorts using healthy controls, and the Caikovska et al. study adds the inverse AAT-RNFL correlation and the inverse AAT-folate in-patient anti-correlation—advancing the systemic biomarker evidence from isolated case-control differences to findings with structural correlates and mechanistic coherence. The H4BIP hypothesis and the KP mechanistic bridge remain well-grounded working hypotheses from a single program requiring external replication and direct cofactor measurement. The fundamental limitations—absence of healthy AH controls, direct H4BIP measurement, AH NAD quantification, and treatment-naive cohort data—are the primary constraints on mechanistic inference from the existing datasets.

Glaucoma in a substantial subset of patients may represent a disease of progressive coenzyme and cofactor failure—H4BIP insufficiency, NAD precursor depletion, and folate deficiency operating simultaneously within a context of low-grade systemic inflammatory activation that drives KMO upregulation and AAT consumption in parallel. If confirmed by direct measurement of these variables in the same treatment-naive patients, this framework would redefine glaucoma management beyond IOP reduction toward multimodal metabolic neuroprotection that is already partially actionable through nicotinamide and B-vitamin supplementation.

## Figures and Tables

**Table 1:** Current intraocular pressure-lowering treatment classes, their mechanisms, and principal metabolic and mitochondrial considerations relevant to the framework of this review.

Drug Class	Example Agents	IOP Mechanism	Key Side Effects	Metabolic/Mitochondrial Relevance
Beta-adrenergic antagonists	Timolol, betaxolol	Reduce aqueous humor production (~20–30%) [6]	Bradycardia, bronchospasm, fatigue	Reduce AH production—may concentrate AH metabolites non-specifically; systemic mitochondrial respiratory effects reported with timolol [7]
Prostaglandin analogues	Latanoprost, bimatoprost	Increase uveoscleral outflow [8]	Iris pigmentation, periorbital fat atrophy, eyelash growth	No direct metabolic pathway relevance; TGF-β interactions potentially relevant to PEXS [9]
Carbonic anhydrase inhibitors	Dorzolamide, brinzolamide	Reduce aqueous production via CA-II inhibition [10]	Metabolic acidosis (systemic); ocular burning (topical)	CA-II inhibition alters AH acid-base balance; systemic CA inhibitors reduce HCO_3_^−^ and may affect mitochondrial CO_2_ handling [11]
Alpha-2 agonists	Brimonidine	Reduce aqueous production; increase uveoscleral outflow [12]	CNS depression (infants); dry mouth; allergy	Brimonidine has independent neuroprotective properties via α2-receptor-mediated BDNF upregulation, relevant to RGC survival [13]
Rho kinase inhibitors	Netarsudil	Increase trabecular outflow; reduce episcleral venous pressure [14]	Conjunctival hyperaemia, cornea verticillata	Novel class with vascular and NO-pathway effects; potentially relevant to arginine/proline and eNOS framework [15]

IOP = intraocular pressure; AH = aqueous humor; CA = carbonic anhydrase; PEXS = pseudoexfoliation syndrome; TGF-β = transforming growth factor-beta; BDNF = brain-derived neurotrophic factor; RGC = retinal ganglion cell; eNOS = endothelial nitric oxide synthase; NO = nitric oxide.

**Table 2:** Cross-condition AH metabolite fingerprints from the Pauls Stradins platform.

Metabolite/Pathway	Glaucoma [24,25]	PEXS [25,27]	Diabetes [25,28]	Interpretation
Phe ↑, Tyr ↑	Yes	Yes	Variable	Non-specific oxidative/inflammatory signal
Trp ↑ (substrate accumulation)	Yes	Yes	No (3-HK ↑ instead)	Upstream block in glaucoma/PEXS; downstream flux in diabetes
KMO ↑ (3-HK/KYN ratio)	Yes	No	Yes	Absent in PEXS despite shared Trp ↑—critical specificity finding
Gln ↑	Yes	Not reported	Not reported	Cross-validated across independent groups [18,22,23]
3-HK ↑ (product elevation)	No	No	Yes	Diabetes-specific KMO saturation—distinguishes from glaucoma
KAT ↓ (SPONCS-dependent)	Yes (confound)	Yes (confound)	Yes (confound)	Universal cataract-severity confound requiring SPONCS covariate [26]

Phe = phenylalanine; Tyr = tyrosine; Trp = tryptophan; KMO = kynurenine monooxygenase; KAT = kynurenine aminotransferase; 3-HK = 3-hydroxykynurenine; KYN = kynurenine; Gln = glutamine; PEXS = pseudoexfoliation syndrome; SPONCS = Simplified Pre-Operative Nuclear Classification Score. Note: aromatic amino acids (Tyr, Phe, Trp) function as potent intrinsic reducers; their elevation in AH may directly modulate local redox status via radical scavenging and electron donation.

## Data Availability

No new data were created or analyzed in this review. Data sharing is not applicable to this article.

## Abbreviations

The following abbreviations are used in this manuscript:

3-HK	3-hydroxykynurenine
AAT	alpha-1-antitrypsin
AH	aqueous humor
ARE	antioxidant response element
H2BIP	7,8-dihydrobiopterin
H4BIP	tetrahydrobiopterin
DHFR	dihydrofolate reductase
DHPR	dihydropteridine reductase
DR	diabetic retinopathy
eNOS	endothelial nitric oxide synthase
FC	fold change
FDR	false discovery rate
GCH1	gene encoding GTP cyclohydrolase I
GCIPL	ganglion cell-inner plexiform layer
GTPCH I	GTP cyclohydrolase I
IDO	indoleamine 2,3-dioxygenase
IFN-γ	interferon-gamma
IL-1β	interleukin-1 beta
IL-6	interleukin-6
IOP	intraocular pressure
iPSC	induced pluripotent stem cell
KAT	kynurenine aminotransferase
KMO	kynurenine monooxygenase
KP	kynurenine pathway
KYN	kynurenine
KYNA	kynurenic acid
LC-HRMS	liquid chromatography high-resolution mass spectrometry
MTHFR	methylenetetrahydrofolate reductase
NAD	nicotinamide adenine dinucleotide
NMN	nicotinamide mononucleotide
NO	nitric oxide
NOS	nitric oxide synthase
Nrf2	nuclear factor erythroid 2-related factor 2
NTG	normal tension glaucoma
OCR	oxygen consumption rate
ONH	optic nerve head
ONOO^−^	peroxynitrite
OHT	ocular hypertension
O_2_•^−^	superoxide
PACG	primary angle-closure glaucoma
PAH	phenylalanine hydroxylase
PBMC	peripheral blood mononuclear cell
PCD	pterin-4a-carbinolamine dehydratase
PEXG	pseudoexfoliative glaucoma
PEXS	pseudoexfoliation syndrome
PGC-1α	peroxisome proliferator-activated receptor gamma coactivator-1 alpha
POAG	primary open-angle glaucoma
RGC	retinal ganglion cell
RNFL	retinal nerve fiber layer
ROS	reactive oxygen species
SERPINA1	serpin family A member 1 gene
SIRT	sirtuin
SPONCS	Simplified Pre-Operative Nuclear Classification Score
SPR	sepiapterin reductase
TGF-β	transforming growth factor-beta
TH	tyrosine hydroxylase
Tfam	mitochondrial transcription factor A
TNF-α	tumor necrosis factor-alpha
TPH	tryptophan hydroxylase
VF	visual field
5-MTHF	5-methyltetrahydrofolate
PCD	4a-carbinolamine dehydratase
